# Genome Sequence of *Bacillus endophyticus* and Analysis of Its Companion Mechanism in the *Ketogulonigenium vulgare-Bacillus* Strain Consortium

**DOI:** 10.1371/journal.pone.0135104

**Published:** 2015-08-06

**Authors:** Nan Jia, Jin Du, Ming-Zhu Ding, Feng Gao, Ying-Jin Yuan

**Affiliations:** 1 Key Laboratory of Systems Bioengineering (Ministry of Education), School of Chemical Engineering and Technology, Tianjin University, Tianjin, 300072, PR China; 2 SynBio Research Platform, Collaborative Innovation Centre of Chemical Science and Engineering (Tianjin), School of Chemical Engineering and Technology, Tianjin University, Tianjin, 300072, PR China; 3 Department of Physics, Tianjin University, Tianjin, 300072, PR China; Huazhong University of Science and Technology, CHINA

## Abstract

*Bacillus* strains have been widely used as the companion strain of *Ketogulonigenium vulgare* in the process of vitamin C fermentation. Different *Bacillus* strains generate different effects on the growth of *K*. *vulgare* and ultimately influence the productivity. First, we identified that *Bacillus endophyticus* Hbe603 was an appropriate strain to cooperate with *K*. *vulgare* and the product conversion rate exceeded 90% in industrial vitamin C fermentation. Here, we report the genome sequencing of the *B*. *endophyticus* Hbe603 industrial companion strain and speculate its possible advantage in the consortium. The circular chromosome of *B*. *endophyticus* Hbe603 has a size of 4.87 Mb with GC content of 36.64% and has the highest similarity with that of *Bacillus megaterium* among all the bacteria with complete genomes. By comparing the distribution of COGs with that of *Bacillus thuringiensis*, *Bacillus cereus* and *B*. *megaterium*, *B*. *endophyticus* has less genes related to cell envelope biogenesis and signal transduction mechanisms, and more genes related to carbohydrate transport and metabolism, energy production and conversion, as well as lipid transport and metabolism. Genome-based functional studies revealed the specific capability of *B*. *endophyticus* in sporulation, transcription regulation, environmental resistance, membrane transportation, extracellular proteins and nutrients synthesis, which would be beneficial for *K*. *vulgare*. In particular, *B*. *endophyticus* lacks the Rap-Phr signal cascade system and, in part, spore coat related proteins. In addition, it has specific pathways for vitamin B12 synthesis and sorbitol metabolism. The genome analysis of the industrial *B*. *endophyticus* will help us understand its cooperative mechanism in the *K*. *vulgare*-*Bacillus* strain consortium to improve the fermentation of vitamin C.

## Introduction

The microbial ecosystem of *Ketogulonigenium vulgare* and *Bacillus* strains has been widely used in the two-steps vitamin C fermentation processes [1]. In bacterial communities, *K*. *vulgare* is responsible for the conversion of sorbose to 2-keto-L-gulonic acid (2-KLG), the precursor of vitamin C. *Bacillus* strains (e.g., *B*. *megaterium*, *B*. *cereus* and *B*. *thuringiensis*) are co-cultured to stimulate the growth of *K*. *vulgare* [2]. Moreover, different 2-KLG yields and productivities were observed in the consortium with different companion strains [3]. Clearly, the varied growth characteristics of the different companion strains might produce different effects on the fermentation process. Researchers are always looking for the best strains to cooperate with *K*. *vulgare*, and we identified that *B*. *endophyticus* Hbe603 is the appropriate strain because the product conversion rate exceeded 90% in industrial vitamin C fermentation. *B*. *endophyticus* is an aerobic, Gram-positive, non-motile, rod-shaped, endospore-forming bacterium, which was first isolated from the inner tissues of cotton plants [4]. It has been extensively applied for promoting plant-growth [5] and decolorizing textile effluents [6]. Knowledge on industrial strains will help us further understand the natural variation and the possible differences among *Bacillus* strains and their communication with *K*. *vulgare*.

The interaction and communication between *Bacillus* strains and *K*. *vulgare* have been investigated by metabolomic and proteomic analysis [7–10]. Further analysis of the genetic makeup and complementation are needed to understand the consortium. Genome sequence analysis could provide further information to distinguish the differences between strains and determinate the symbiotic relationship between the microorganisms at the gene level. For example, the genome analysis of the UCYN-A *cyanobacteria* found the absence of numerous major metabolic pathways and the necessary electron transport capacity to generate energy, which suggests that this strain must depend on other organisms to obtain critical nutrients [11]. The genome analysis of *Syntrophus aciditrophicus* provided a glimpse on its composition and identified that the electron transfer and energy transducing systems were used for the syntrophic life [12].

Currently, the genome-wide research on the *B*. *endophyticus* strain is still scarce and only one draft genome sequence of *B*. *endophyticus* 2102 has been published [13]. Here, we report a 4.87 Mb circular chromosome of *B*. *endophyticus* Hbe603, which is used as the companion strain in the vitamin C industrial fermentation process. Through the comparative genome analysis of *B*. *endophyticus* with other species, we found evidence of its special features, such as sporulation, transcription regulation, environmental resistance, membrane transportation, extracellular protein release and nutrients synthesis. Likewise, we speculate its companion mechanism in the *K*. *vulgare*-*Bacillus* strain consortium.

## Materials and Methods

### Strains and cultivation conditions

The *B*. *endophyticus* HBe603 strain was cultured in 250 mL flasks with 50 mL of seed medium (30°C, 250 rpm) supplied with D-sorbitol (2%) for 35 h to determine the sporulation and growth curve. The seed medium contains 3 g/L beef extract, 3 g/L yeast powder, 3 g/L corn steep liquor, 0.2 g/L MgSO_4_, 1 g/L KH_2_PO_4_, 1 g/L urea and 10 g/L peptone.

### Measurement of cell density and analysis of D-sorbitol

The cell density was measured as optical density at 600 nm (OD_600_) with a spectrophotometer, and cells were observed under a phase contrast microscope. D-sorbitol in the broth was analyzed by HPLC (Waters Corp., Massachusetts, USA) with a refractive index detector. In addition, 5 mM H_2_SO_4_ was used as the eluent on the Aminex HPX-87H column (BioRad, CA) at 65°C with a flow rate of 0.6 mL/min.

### DNA extraction and quality control

A genome sample was extracted using a Bacteria DNA Kit (QIAGEN) according to the manufacturer’s instructions. Briefly, cells were lysed with lysozyme and treated with proteinase K. The lysate was then treated with 20% sodium dodecyl sulfate and cetyltrimethylammonium bromide. Afterwards, the DNA was extracted with phenol/ chloroform. Then, the DNA was precipitated with ethanol and sodium acetate and it was washed twice with 70% ethanol. Each sample was treated with RNaseA at 37°C for 30 min to allow RNA degradation. The quality of the DNA was assessed by spectrophotometer and gel electrophoresis. DNA samples with a 260/280 nm absorbance ratio of 1.8–2.0 and a 260/230 nm absorbance ratio of 2.0–2.2 were considered pure. Only high molecular weight pure DNA samples were used for the construction of the library and sequencing.

### Sequencing and assembly

Each SMART bell sequencing library was constructed using 500 ng size-selected DNA with the Pacific Biosciences DNA Template Prep Kit 2.0. The binding of SMRT bell templates to polymerases was conducted using the DNA/Polymerase Binding Kit P5 and v2 primers. Sequencing was carried out on the Pacific Bioscience RS II platform using C3 reagents with 120 min movies. The .h5 files resulting from the PacBio sequencing were used directly for the assembly process. The raw reads were processed into subreads by removing the adaptors and filtered using SMRT Analysis 2.2 (http://www.pacb.com/devnet/) with minSubReadLength = 500 and readScore > 0.75. The filtered subreads were used in the HGAP assembly process. An in-house Perl script was used to calculate the distribution of subread lengths and identify the range of lengths that would give a coverage around 10. These length values were chosen as the seed length in the HGAP assembly process [14]. For *B*. *endophyticus* HBe603, seed length 6K-14K was chosen. A separate assembly process was done for each seed length. The HGAP assembly process was done as follows: 1) Reads shorter than the seed length were aligned to the longer reads using BLASR [15]. The errors on the long reads were corrected using the aligned reads; 2) The high quality corrected reads were assembled based on overlapping sequences to obtain a draft assembly; 3) All the reads were mapped to the draft assembly, which polished the assembly to obtain the final genomic sequence. The HGAP parameters used were genomeSize = 5000000, xCoverge = 15, defaultFrgMinLen = 500, ovlErrorRate = 0.06, ovlMinLen = 40, merSize = 14. The seed length that gave the least contigs were chosen as the final assembly. The assembled sequences were checked by BLAST to the NCBI database whether the contigs show similarity to known genomes or plasmids. For circular chromosome, we ran BLAST against itself to identify the redundant sequences at the end. The redundant sequences from the 3’ end were clipped and the connected part was examined by PCR.

### Genome annotation and bioinformatics analysis

The *de novo* gene prediction of the genome sequence was performed by GeneMarkS [16]. The gene function was annotated by using BLAST [17] against Kyoto Encyclopedia of Genes and Genomes database KEGG [18], SWISS-PROT [19] and Clusters of Orthologous Groups of proteins database (COG) [20]. The tRNAs and rRNAs were predicted by tRNAscan-SE [21] and RNAmmer [22], respectively. The essential genes were predicted by ZCURVE 3.0 [23] and DEG 10 [24], respectively. The subcellular location of proteins and the signal peptides were commented by PSORT [25] and SignalP 4.0 [26], respectively. The origin of replication (*oriC*) and putative DnaA boxes were identified using Ori-Finder [27]. CVTree, a whole genome-based, alignment-free composition vector (CV) method was performed for the phylogenetic analysis [28], and a phylogenetic tree was generated using the MEGA program [29]. The GC-Profile was used to compute the GC content variation in DNA sequences and predict the genomic islands [30]. The circular chromosome map was created using the program CGView [31]. The sequence similarity was analyzed using ACT (the Artemis Comparison Tool) [32].

### Nucleotide sequence accession numbers

The sequence of the *B*. *endophyticus* Hbe603 chromosome has been deposited in GenBank under the accession number CP011974.

## Results and Discussion

### General genomic properties

**Fig 1:**
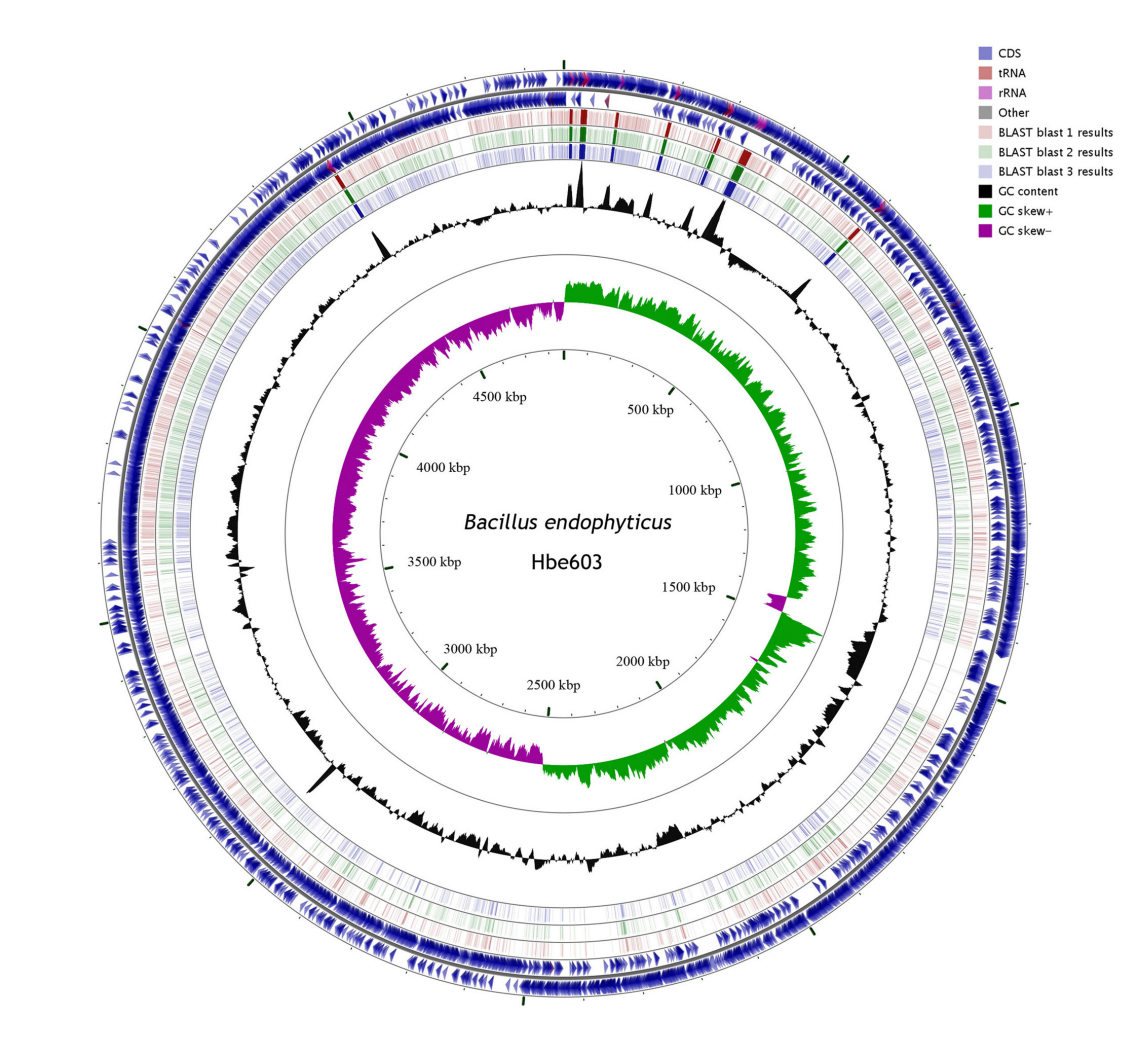
Circular genome visualization of *B*. *endophyticus* Hbe603. Circles from the outside to the inside show the positions of protein-coding genes (blue), tRNA genes (red) and rRNA genes (pink) on the positive (circle 1), and negative (circle 2) strands. Circles 3–5 show the positions of BLAST hits detected through blastx comparisons of *B*. *endophyticus* Hbe603 against *B*. *megaterium* QM B1551 (circle 3), *B*. *megaterium* DSM 319 (circle 4) and *B*. *megaterium* WSH-002 (circle 5). The height of the shading in the BLAST results rings is proportional to the percentage of identity of the hit. Circles 6 and 7 show plots of GC content and GC skew plotted as the deviation from the average for the entire sequence.

**Fig 2:**
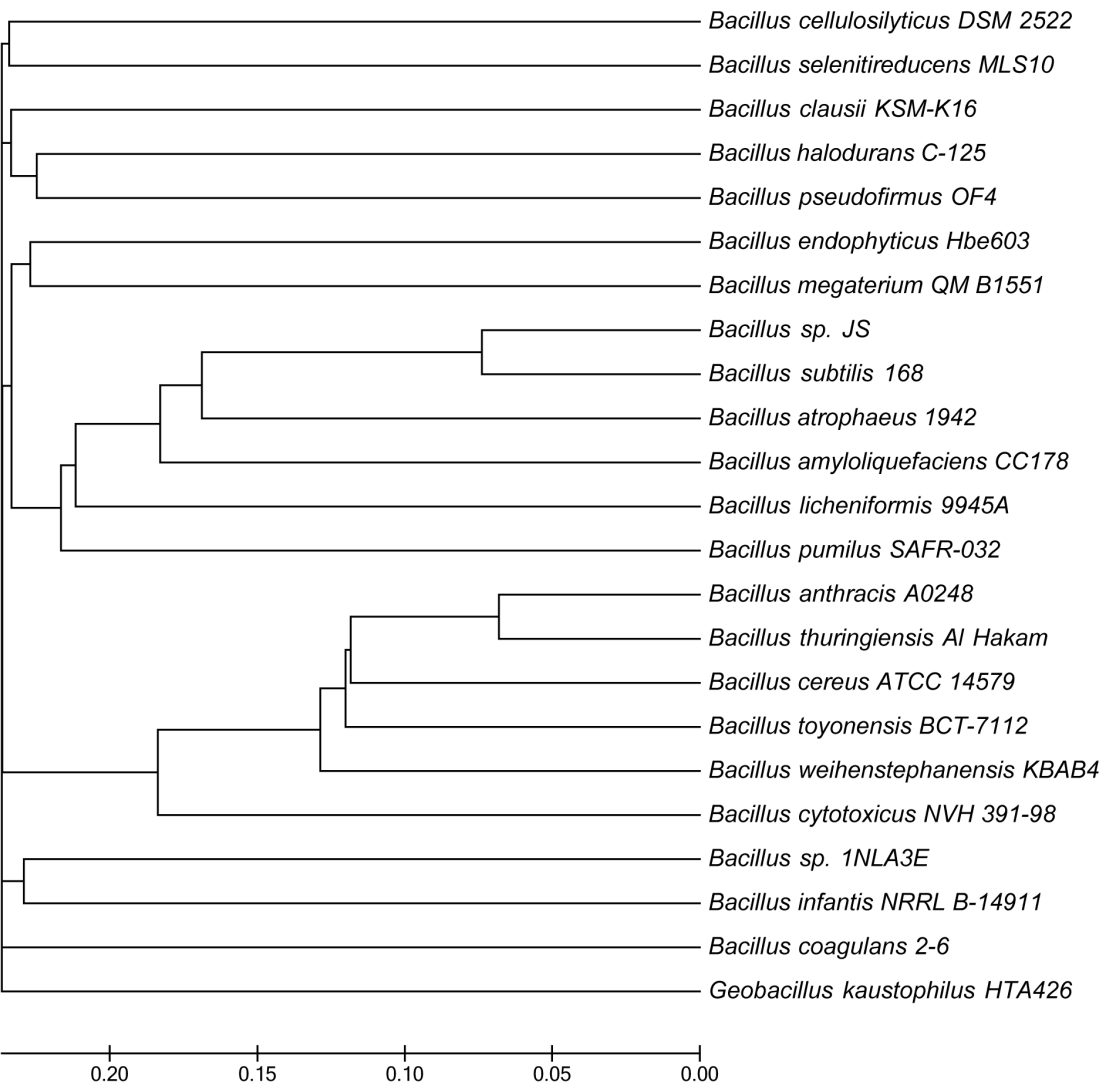
Phylogenetic analysis of *B*. *endophyticus* Hbe603 with other species. The phylogenetic tree of *B*. *endophyticus* Hbe603 was constructed using CVTree with parameters K = 6 and Type = aa. The neighbor-joining tree was constructed using the MEGA5 program based on the CVTree results. Note that *Geobacillus kaustophilus* HTA426 was included as an outgroup.

**Fig 3:**
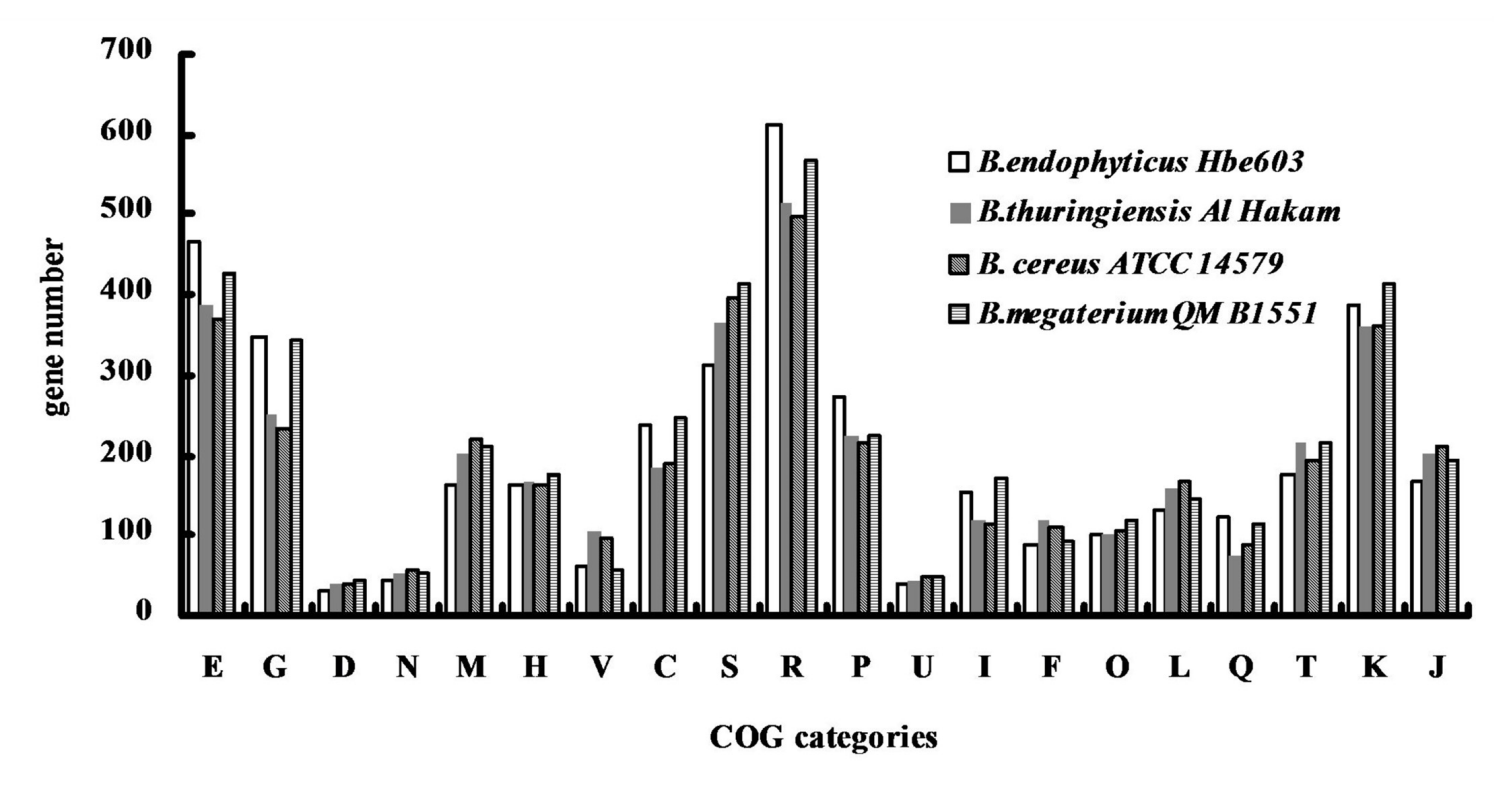
COG analysis of *B*. *endophyticus* Hbe603 with other *Bacillus* species. The information presented here corresponds to the original annotation. Alterations could occur due to possible updates. The information is in accordance to the genome information provided in the corresponding NCBI .gbk files. COG designations are described as follows: C, Energy production and conversion; D, Cell division and chromosome partitioning; E, Amino acid transport and metabolism; F, Nucleotide transport and metabolism; G, Carbohydrate transport and metabolism; H, Coenzyme metabolism; I, Lipid metabolism; J, Translation, ribosomal structure and biogenesis; K, Transcription; L, DNA replication, recombination, and repair; M, Cell envelope biogenesis, outer membrane; N, Cell motility and secretion; O, Posttranslational modification, protein turnover, chaperones; P, Inorganic ion transport and metabolism; Q, Secondary metabolite biosynthesis, transport and catabolism; R, General function prediction only; S, Function unknown; T, Signal transduction mechanisms; U, Intracellular trafficking and secretion; V, Defense mechanisms.

The *B*. *endophyticus* Hbe603 chromosome is 4.87 Mb with GC content of 36.64% and contains 5,038 annotated genes (Fig 1, Table 1 and S1 Table). We detected four prophages in *B*. *endophyticus* Hbe603 using PHAge Search Tool (PHAST) [33] (S1 Fig). In the four prophages, most of the small proteins are annotated as hypothetical proteins that may play important roles in response to specific environmental stresses and host adaptation [34]. The other functional genes encode 59 phage-like proteins, two phage integrases and two transposases. Besides the prophage regions, the complete chromosome sequence of *B*. *endophyticus* Hbe603 has the high consistency with the draft sequence of *B*. *endophyticus* 2102 (S2 Fig). In addition to the published companion strains *B*. *thuringiensis* [35], *B*. *cereus* [36] and *B*. *megaterium* [37], we identified that *B*. *endophyticus* Hbe603 is the appropriate strain to cooperate with *K*. *vulgare* and the product conversion rate exceeded 90% in industrial vitamin C fermentation. Through a whole genome-based phylogenetic analysis, we can conclude that *B*. *endophyticus* is a closer companion strain to *B*. *megaterium* QM B1551 [38] than *B*. *cereus* ATCC 14579 [39] and *B*. *thuringiensis* Al Hakam [40] (Fig 2). By comparing the distribution of COG classification among the four strains, we could assess their gene function distributions and their genetic relationships (Fig 3). In the *B*. *endophyticus* Hbe603 genome, the number of genes related to cell envelope biogenesis (M) and signal transduction mechanisms (T) is lower than that in the other three strains, while the number of genes related to carbohydrate transport and metabolism (G), energy production and conversion (C) and lipid transport and metabolism (I) is similar to that in *B*. *megaterium* and higher than those in the other two strains (S2 Table). Overall, *B*. *endophyticus* Hbe603 has unique properties with regards to protein function and is more similar to *B*. *megaterium* than the other strains. Interestingly, *B*. *megaterium* has been used for industrial vitamin C production in Jiangshan Pharmaceutical Co. Ltd., China [41]. Since both strains can become industrial companion strains, they presumably show common characteristics to have a better interaction with *K*. *vulgare*.

**Table 1:** General features of the genome sequence of *B*. *endophyticus* Hbe603 and 2102.

Feature	*B*. *endophyticus* Hbe603	*B*. *endophyticus* 2102
**Length of sequence (bp)**	4865574	5107189
**G+C content (%)**	36.64	36.40
**Protein-coding gene number**	5038	5186
**rRNA number**	33	7
**tRNA number**	81	49

### Genetic analysis of *B*. *endophyticus*’ companion effect on *K*. *vulgare*

#### Genes related to the sporulation process

Several researchers have indicated that the spore stability of *Bacillus* strains plays an important role in stimulating the propagation of *K*. *vulgare* and the accumulation of 2-KLG [9,42]. During the process of the spore formation, cells burst and release intracellular metabolites that significantly promote the growth of *K*. *vulgare*. Thus, we analyzed the genes related to the different sporulation stages to understand the sporulation process and the regulation mechanism of *B*. *endophyticus* Hbe603 (S3 Table). Current research on the process and mechanism of sporulation mainly focus on the model strain *B*. *subtilis*. The lifecycle of *B*. *subtilis* is generally summarized in seven steps: vegetation (stage zero and I), stage II, stage III, stage IV, stage V, spore maturation (stage VI and VII) and spore germination [43,44]. About 140 genes related to the sporulation cycle were identified by the genome annotation of *B*. *endophyticus* Hbe603, and most of them have a high similarity to those in *B*. *subtilis*. These data confirm the complete sporulation ability of *B*. *endophyticus* Hbe603. At the initial stage of the spore formation, *spo0H* and *spo0A* encode a related regulatory factor, which is capable of regulating the cell growth and initializing the spore formation [45]. The histidine kinases KinA, KinD and KinE [46,47] respond to environmental stimulation and then phosphorylate Spo0A to form a two-component sensing system until the spore formation process begins. In addition, the genes related to spore coat formation in *B*. *endophyticus* Hbe603 were compared with those in other *Bacillus* strains to analyze the properties of the spores. Among the genes related to the outside spore coat, *B*. *endophyticus* Hbe603 only has *cotA* and *cotE*, and lacks *cotB*, *cotC*, *cotG*, *cotM*, *cotO*, *cotY* and *ytxO*, which are annotated in *B*. *subtilis*. *B*. *megaterium* only has *cotB* and *cotE*, and many similarities exist between the two species with regards to the structure of the outside spore coat. Among the inside spore coat genes, *B*. *endophyticus* Hbe603 has *cotD*, *cotJA*, *cotJB*, *cotJC*, *cotF*, *yutH*, *yaaH*, *yheC* and *yheD*, and lacks *cotH*, *ymaG*, *cotT*, *yxeE*, *yeeK* and *ysnD*, which are annotated in *B*. *subtilis*. In addition, there are three operons, *cgeAB*, *cgeCDE* and *spsABCDEFGHIJKL*, which encode a glycosyl transferase in *B*. *subtilis* and participate in the spore coat glycosylation [48]. *B*. *thuringiensis* lacks *spsD*, and *B*. *cereus* only has *spsI*, *spsJ* and *spsK* [49]. *B*. *endophyticus* Hbe603 and *B*. *megaterium* completely lack these three operons, and that deficiency may improve the hydrophobicity of spores and their gathering ability, thus, enhancing the affinity between spores and nonspecific surfaces [50]. *B*. *endophyticus* Hbe603 and *B*. *megaterium* lack related genes rendering this type of spore characteristics potential beneficial effects in synergistic actions.

#### Genes related to the regulation of transcription

Compared to *K*. *vulgare*, companion *Bacillus* strains have a stronger ability of responding and adapting to environmental changes, and the transcriptional regulation system plays an important role. *B*. *endophyticus* Hbe603 has nearly 300 genes related to regulation, including 17 sigma factor encoding genes (Table 2). As a general regulatory factor, sigma-B controls a large number of pressure-responsive related proteins. Previous research has reported two types of regulation mechanisms of sigma-B in *Bacillus* strains, i.e., that of *B*. *subtilis* [51] and that of *B*. *cereus* [49]. The genes related to the regulation of sigma-B in *B*. *endophyticus* Hbe603 are similar to those in *B*. *Subtilis*. During unstressed conditions, the anti-sigma factor RsbW directly combines with sigma-B, while the anti-anti-sigma factor RsbV is in the phosphorylated state and is unable to combine with RsbW [52]. In addition, RsbU dephosphorylates RsbV and releases sigma-B to initiate its transcriptional activity at ambient state. Likewise, a series of cascade factors can regulate the activity of RsbU phosphorylation, such as RsbX, RsbT, RsbS and the RsbR family of proteins (RsbRA, RsbRB and RsbRD). However, we could not find the regulatory factor RsbP in *B*. *endophyticus* Hbe603, which is responsible for the energy pressure in *B*. *subtilis* [53]. The sigma factor ECF (extracytoplasmic function) can induct extracellular environment stress and regulate the signal response. A total of seven related genes were detected in *B*. *endophyticus* Hbe603. Similarly, *B*. *subtilis* has seven genes, *B*. *cereus* has ten and *B*. *thuringiensis* has thirteen genes [49]. Among the seven sigma factors, we found two sigma-M factors, which can respond to high salt concentration and regulate the strain to adapt to high osmotic pressures in the environment [54]. Sigma-C, Sigma-V, Sigma-X and Sigma-W respond to temperature, lysozyme, iron and bacteriocin toxins, respectively. In addition of being important regulation factors, the Rap family proteins commonly exist in *Bacillus* strains and are combined with the signal peptide Phr to form the Rap-Phr signal cascade system [55]. This signal cascade system responds to cell density and regulates the initiation of sporulation. *B*. *subtilis* contains eleven Rap-encoding genes and seven Phr-encoding genes, and the number of related genes is slightly lower in *B*. *cereus* and *B*. *thuringiensis*. Nonetheless, only one related protein PhrA was detected in *B*. *endophyticus* Hbe603 and it has a high similarity with that of *Agrobacterium tumefaciens*. Hence, *B*. *endophyticus* Hbe603 may contain other pathways to respond to cell density and to initiate spore formation. These characteristics might be attributed to its specific communication pattern and its better companion ability.

**Table 2:** Predicted sigma factors in *B*. *endophyticus* Hbe603.

Locus	Sigma Factor	Annotation
*Be_0112*	sigma-H	sporulation and competence, cytochrome biogenesis, generation of potential nutrient sources, transport, and cell wall metabolism
*Be_0199*	sigma-W	resistance to bacteriocins and cell envelope- damaging compounds
*Be_0276*	sigma-B	general stress response
*Be_0926*	sigam-I	control of a class of heat shock genes
*Be_1343*	sigma-C	response to temperature upshift
*Be_1353 Be_3294*	sigam-M	response to high concentration of salt
*Be_3347 Be_4015*	sigma-X	response to iron
*Be_3821*	sigma-D	regulation of flagella, motility, chemotaxis and autolysis
*Be_3924*	sigma-G	transcription of sporulation genes
*Be_3925*	sigma-E	transcription of sporulation genes
*Be_4047*	sigma-F	transcription of sporulation genes
*Be_4212*	sigma-A	major sigma factor of RNA polymerase
*Be_4265*	sigma-K	RNA polymerase sporulation-specific sigma factor
*Be_4753*	sigma-L	utilization of arginin, acetoin and fructose, required for cold adaptation
*Be_4938*	sigma-V	response to lysozyme

#### Genes related to Environmental resistance

Previous research identified that reduced glutathione could significantly improve the growth of *K*. *vulgare* [56], and a proteomic analysis revealed its high demand for antioxidant protection [10]. *B*. *endophyticus* Hbe603 has a strong environmental resistance and relieves the stress of *K*. *vulgare* [9]. *B*. *endophyticus* Hbe603 contains a complete heat shock system, Clp, which is associated with high temperature tolerance. That system contains the chaperone ClpB, ATPase subunit ClpE [57], ClpP, ClpX [58], protein degradation subunits ClpY and ClpQ, and the CtsR global response protein [59]. Moreover, *B*. *endophyticus* Hbe603 has eight Na^+^/H^+^ antiporter related genes, the cluster *mrpABCDEFG* and *nhaC*. The *mrp* complex contains seven Na^+^/H^+^ antiporter subunits, which are associated with cell tolerance in alkaline environments. This complex responds to proton motive force in the cell membrane, where H^+^ is transported to the inside of the cells, and Na^+^ is pumped out [60]. The NhaC protein plays an important role in maintaining a stable pH environment, and it has a high similarity with that of *Bacillus pseudofirmus* OF4. This strain is an alkali resistant microorganism that can grow in pH ranging from 7.5 to 11.4 [61]. In addition, the *yhaU/khtT* gene clusters were detected in *B*. *endophyticus* Hbe603 that encode K^+^/H^+^ antiporters and pump out K^+^ to maintain a stable pH in alkaline environments [61]. The ability of *B*. *endophyticus* Hbe603 to adapt the alkaline environment of the industrial fermentation process might be related with the above mechanism. Microorganisms also need to absorb large quantities of K^+^ to maintain an osmotic balance in a high permeability pressure environment. *B*. *endophyticus* Hbe603 has the complete Ktr system to perform this function, which includes the *ktrAB*, *ktrC* and *ktrD* operon [62]. Several studies have shown that *B*. *megaterium* increases the proline synthesis pathway in high salt conditions [63]. Accordingly, the *proHJA* gene cluster is present in the *B*. *endophyticus* Hbe603 genome and has the ability to complete the synthesis of proline. In addition, glycine betaine is an effective protective agent against osmotic pressure. Interestingly, *B*. *endophyticus* Hbe603 contains two copies of glycine betaine synthetic enzymes GbsA and a GbsB, two copies of the glycine betaine transporter OpuD, and two operons encoding the glycine betaine/choline ABC transporter. Based on this complex system, *B*. *endophyticus* Hbe603 could be adapted to highly variable environments.

#### Genes related to the membrane transport system

**Fig 4:**
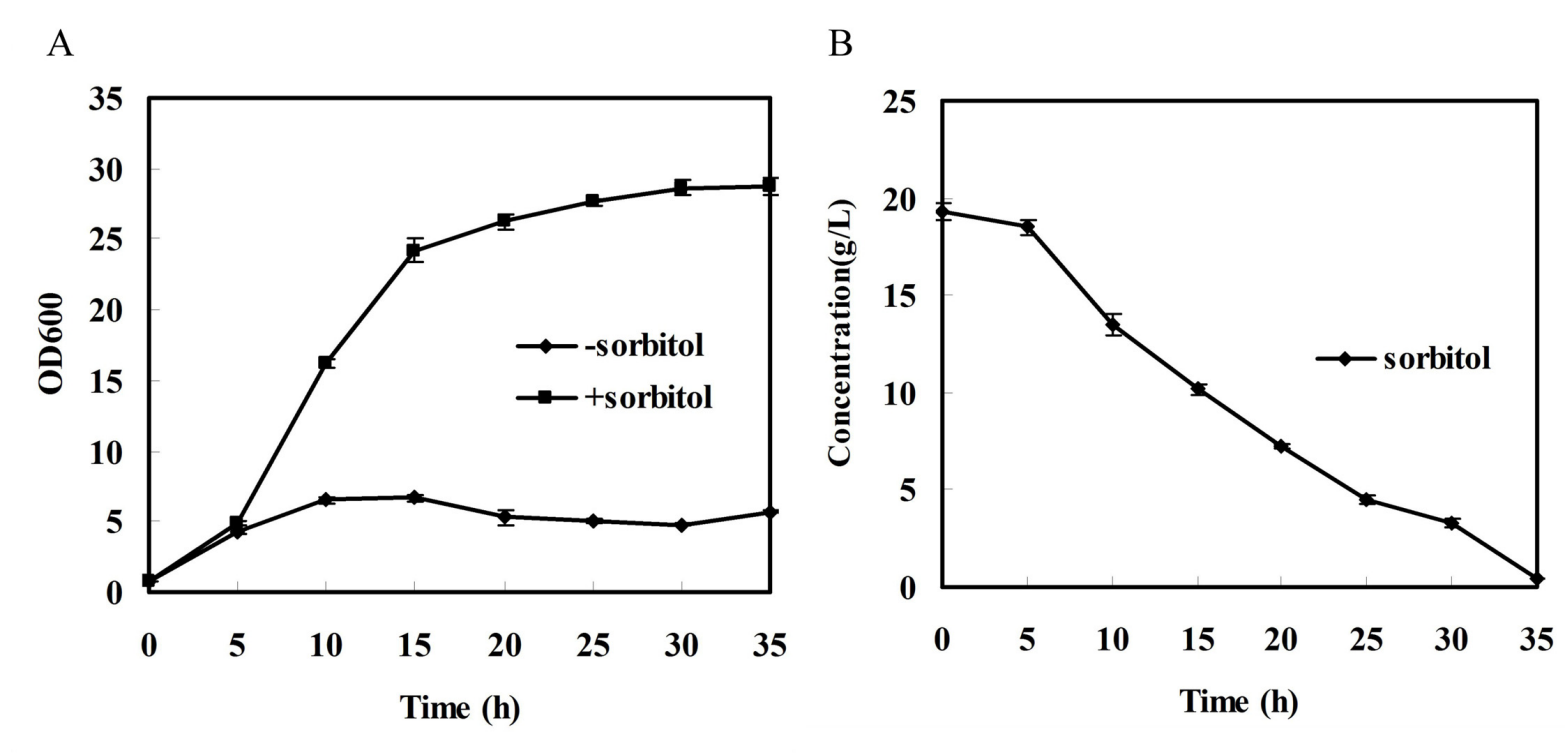
Growth features of the *B*. *endophyticus* Hbe603 strain. A). Growth curve the *B*. *endophyticus* Hbe603 strain grown in seed medium with D-sorbitol (2%). The Y axis represents the average OD_600_ of triplicate bacterial cultures at each time point. B). Extracellular concentration of D-sorbitol. Data are averages of three independent experiments.

The metabolic cooperation in the *K*. *vulgare-B*. *megaterium* consortium has been investigated by cultivating them in the same soft agar plate [64]. We found that *B*. *megaterium* swarmed along the trace of *K*. *vulgare* on the agar plate. A metabolomics analysis has detected a number of metabolites exchange among *K*. *vulgare* and the *Bacillus* strain [8], where the transport system of the membranes plays an important role [65]. *B*. *endophyticus* Hbe603 contains 31 phosphotransferase system (PTS) related genes, which are used for carbohydrate transportation. That number of genes is greater than those in *B*. *subtilis* (25 genes), *B*. *cereus* (18 genes) and *B*. *thuringiensis* (20 genes) [49]. The phosphotransferase system of *B*. *endophyticus* Hbe603 includes three copies of the Crh catabolite repression protein (HPr- like protein) [66], HPr kinase PtsH [67] and HprK [68]. Other proteins are included in the Glc, Lac, Fru, Man and other families (Table 3). It is interesting to remark that *B*. *endophyticus* Hbe603 shows a good growth on seed medium supplied with D-sorbitol (2%) as the sole source of carbon and energy (Fig 4). We annotated the D-sorbitol dehydrogenases and a glucitol/sorbitol-specific transport protein adjacent to it. Furthermore, Sorbose reductase is also annotated and has a high similarity with that of *Candida albicans*. We speculate that the reductase may react with D-sorbitol as well. As the substrate of vitamin C fermentation, D-sorbitol can be consumed by *B*. *endophyticus* and may have an important influence on the final conversion rate. Hence, further research on these enzymes will be important to facilitate molecular modifications. Moreover, *B*. *endophyticus* Hbe603 contains almost 130 ABC transporter related proteins that are mainly used for transportation of peptides (15 proteins), amino acids (15 proteins), ions (35 proteins) and phosphate (8 proteins). In addition, we found 30 uncharacterized ABC transporters, which probably contributes to bacterial drug or antibiotic resistance [69].

**Table 3:** Predicted genes related to PTS system in *B*. *endophyticus* Hbe603.

Locus	Gene	Annotation
*Be_0244*	*mtlA*	PTS system mannitol-specific IICBA component
*Be_0453*	*nagE*	PTS system N-acetylglucosamine-specific IICBA component
*Be_0832*	*treP*	PTS system trehalose-specific IIBC component
*Be_0997*	*glcT*	PtsGHI operon antiterminator
*Be_0998*	*ptsG*	PTS system glucose-specific IICBA component
*Be_0999*	*ptsG*	PTS system glucose-specific IICBA component
*Be_1000*	*ptsH*	Phosphocarrier protein HPr
*Be_1001*	*ptsI*	Phosphoenolpyruvate-protein phosphotransferase
*Be_1246*	*nagP*	PTS system N-acetylglucosamine-specific IICB component
*Be_1317*	*ybbF*	Putative PTS system IIBC component
*Be_1743*	*manP*	PTS system mannose-specific IIBCA component
*Be_2273*	*manP*	PTS system mannose-specific IIBCA component
*Be_2274*	*frwC*	Fructose-like permease IIC component
*Be_2275*	*frwB*	Fructose-like phosphotransferase enzyme IIB component
*Be_2328*	*gatC*	Galactitol permease IIC component
*Be_2380*	*crh*	HPr-like protein Crh
*Be_2623*	*crr*	Glucose-specific phosphotransferase enzyme IIA component
*Be_2626*	*malP*	PTS system maltose-specific IICB component
*Be_2874*	*crh*	HPr-like protein Crh
*Be_2978*	*scrA*	PTS system sucrose-specific IIBCA component
*Be_3022*	*exp5*	PTS system glucose-specific EIICBA component
*Be_3428*	*fruA*	PTS system fructose-specific IIABC component
*Be_3476*	*bglF*	PTS system beta-glucoside-specific IIBCA component
*Be_3535*	*manZ*	Mannose permease IID componen
*Be_3536*	*sorA*	Sorbose permease IIC component
*Be_3537*	*M6_Spy0801*	Probable phosphotransferase enzyme IIB component
*Be_3538*	*manX*	PTS system mannose-specific EIIAB component
*Be_4035*	*licA*	Lichenan-specific phosphotransferase enzyme IIA component
*Be_4036*	*licC*	Lichenan permease IIC component
*Be_4037*	*licB*	Lichenan-specific phosphotransferase enzyme IIB component
*Be_4756*	*crh*	HPr-like protein Crh

#### Proteins released into the extracellular environment

A previous study has found that two extracellular proteins of *B*. *megaterium* can promote cell growth and acid production of *K*. *vulgare*. Their molecular weights are 30~50kD and more than 100kD, respectively [70]. With the help of protein localization analysis, the proteins that *B*. *endophyticus* Hbe603 released into the extracellular environment were detected. In addition to the sporulation and flagellar related proteins, we found extracellular esterase, aminopeptidase and polysaccharide deacetylase, which can digest large molecular substances in the environment of *K*. *vulgare*. Additionally, two copies of superoxide dismutase were annotated, which can remove superoxide and protect *K*. *vulgare* from oxidative injury.

#### Genes related to nutrients synthesis

**Fig 5:**
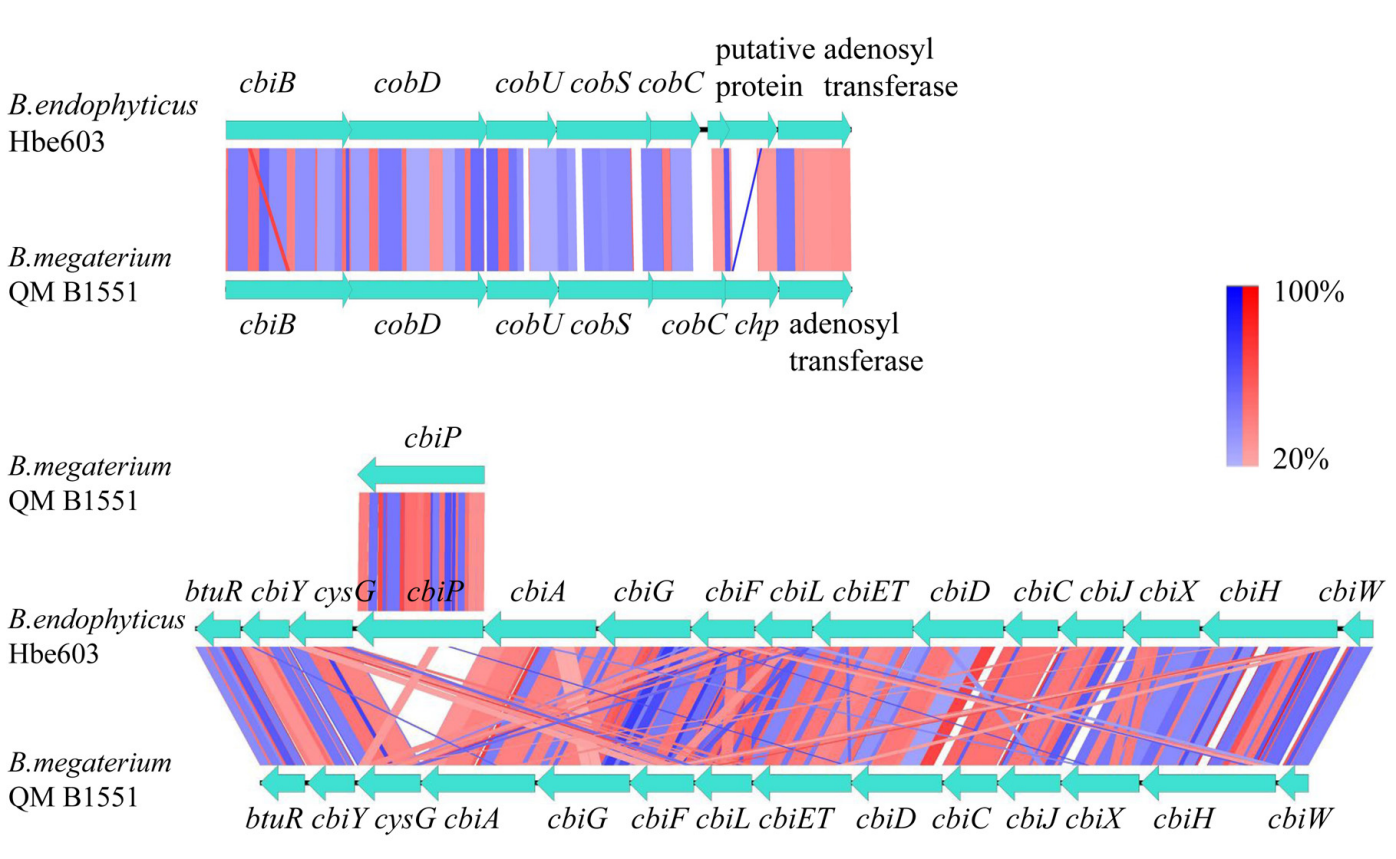
Schematic representation of the genes related to vitamin B12 synthesis in *B*. *endophyticus* Hbe603 by Easyfig. Blue bars represent the forward and reverse strands of DNA with CDSs marked as arrows. The scale is marked in base pairs. The red bars represent normal tblastx matches. Inverted matches are colored in blue, and the depth of shading is indicative of the percentage blast match.

Previously, the metabolic model of *K*. *vulgare* was constructed on a genome-scale [71]. *K*. *vulgare* lacks genes for several pathways such as central metabolism, amino acids metabolism, fatty acids metabolism and vitamins biosynthesis, which might actually impede its growth. Previous studies showed that the addition of L-cysteine to a flask culture of *K*. *vulgare* increased cell growth, 2-KLG titer and the intracellular level of coenzyme A by 25.6%, 35.8%, and 44.7%, respectively [72]. Moreover, the addition of L-glycine, L-proline, L-threonine, L- isoleucine and gelatine increased the 2-KLG productivity by 20.4%, 17.2%, 7.2%, 11.8% and 23.4%, respectively [73]. *B*. *endophyticus* Hbe603 has a relative complete metabolic capacity involved in the supply of amino acids for *K*. *vulgare*, especially L-glycine, L-cysteine, L-methionine, L-tryptophan that *K*. *vulgare* cannot synthesize by itself [74]. In addition, a previous study has shown that *K*. *vulgare* cannot synthesize many B vitamins by itself [74]. We found that *B*. *endophyticus* Hbe603 has vitamin synthesis pathways for B1, B2, B3, B5, B6, B7, B9 and B12, which could be supplied to *K*. *vulgare*. As one of the first biotechnological vitamin B12 producers, *B*. *megaterium* has two distinct and an isolated *cbiP* gene to construct the whole vitamin B12 synthetic pathway [38,75]. *B*. *endophyticus* Hbe603 also has these two distinct genes, but they differ in where the *cbiP* (also called *cobQ*) is inserted. The schematic of genes related to the synthesis of vitamin B12 in *B*. *endophyticus* Hbe603 is presented by Easyfig [76] (Fig 5). Further studies will detect the effect of vitamin B12 production on this genetic difference, and *B*. *endophyticus* is expected to be a suitable engineered strain for the production of vitamin B12. Several cofactors are also supplied by *Bacillus* strains to *K*. *vulgare* in co-culture conditions [71], and we found numerous oxidoreductase-like proteins in *B*. *endophyticus* for the transfer of electrons generated in the cytoplasm. Five putative ferredoxins, two flavodoxins, ten thioredoxins, nine putative nitroreductases, four NADH:flavin oxidoreductases, and 16 quinol/ubiquinol oxidase were annotated in *B*. *endophyticus*. Overall, *B*. *endophyticus* Hbe603 has a relative complete metabolic capacity for the supply of amino acids, vitamins and cofactors for *K*. *vulgare*.

**Fig 6:**
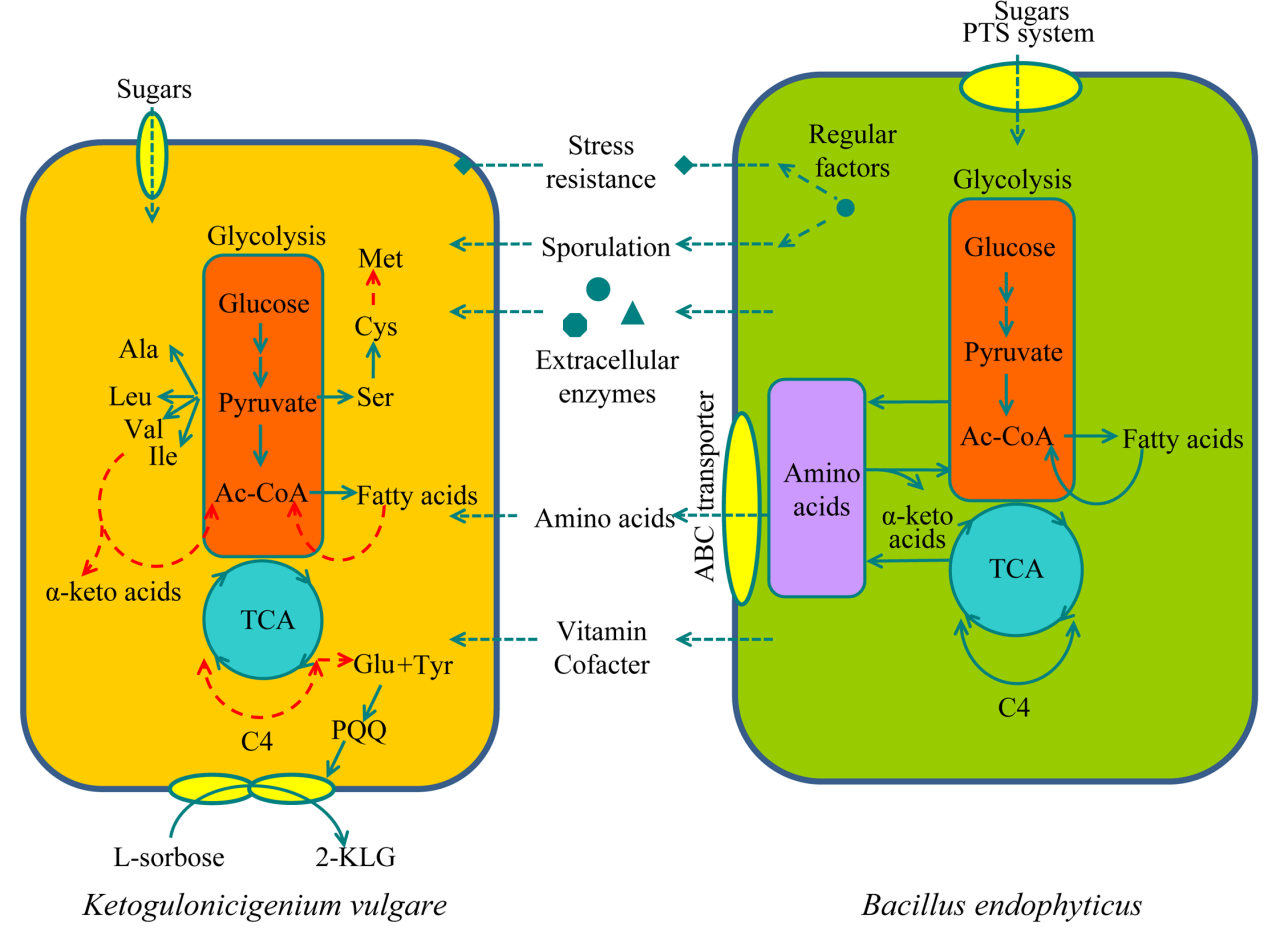
Schematic representation of the interaction and communication between *K*. *vulgare* Y25 and *B*. *endophyticus* Hbe603 at a genome-wide scale. The red dashed line represents the defected pathways in *K*. *vulgare*.

The schematic of *B*. *endophyticus*’ companion mechanism in *K*. *vulgare*-*Bacillus* strain consortium is presented in Fig 6 *B*. *endophyticus* Hbe603 has complex transcriptional regulation systems combined with its ability for spore formation and stress resistance. In addition, *B*. *endophyticus* Hbe603 has rich ABC transporters and proteins related to the PTS system for specific substrate transportation and communication with *K*. *vulgare* at a metabolic level. Likewise, the proteins that *B*. *endophyticus* Hbe603 releases into the extracellular environment may digest large molecular substances and remove superoxide for *K*. *vulgare*. With the sporulation process, *B*. *endophyticus* Hbe603 further releases abundant nutrients (amino acids, vitamins and cofactors) for the growth and the 2-KLG production of *K*. *vulgare*. *B*. *endophyticus* Hbe603 lacks the Rap-Phr signal cascade system and partly spore coat related proteins. In contrast, *B*. *endophyticus* Hbe603 has specific pathways for vitamin B12 synthesis and sorbitol metabolism. Overall, *B*. *endophyticus* provides essential functions that *K*. *vulgare* lacks to reach its maximum growth rate and acts as an alternative source of environmental nutrients in the consortium.

## Conclusions

In summary, we report the chromosome sequence of *B*. *endophyticus* Hbe603 and its annotation, which provide a better-defined genetic background for gene expression and regulation mechanism studies, especially a genome scale metabolic network construction. This comparative genome analysis provides the species-specific characters of *B*. *endophyticus* Hbe603 with respect to other *Bacillus* strains. The corresponding genome analysis will have an enormous impact on our understanding of *K*. *vulgare*-*Bacillus* strain consortium and will help us find more appropriate companion strain in the future.

## Supporting Information

S1 Fig Schematic representing the prophages of *B*. *endophyticus* Hbe603. (TIF)

S2 Fig Comparisons of the sequence similarity between *B*. *endophyticus* Hbe603 and *B*. *endophyticus* 2102 with the Artemis Comparison Tool. (TIF)

S1 Table Genome annotation of *B*. *endophyticus* Hbe603. (XLSX)

S2 Table COG category distribution of *B*. *endophyticus* Hbe603. (DOC)

S3 Table Predicted genes related to sporulation in *B*. *endophyticus* Hbe603. (DOC)
