# Supplementary figures and images for: Genome Sequence of Bacillus endophyticus and Analysis of Its Companion Mechanism in the Ketogulonigenium vulgare-Bacillus Strain Consortium
Source: PLoS One. 2015 Aug 6;10(8):e0135104. doi: 10.1371/journal.pone.0135104 (PMC4527741; doi:10.1371/journal.pone.0135104)

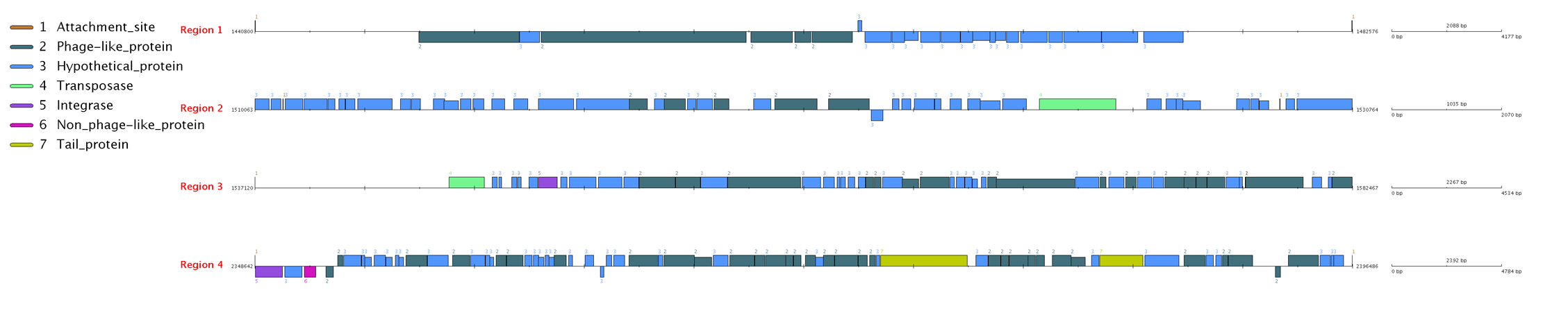

Supplement: S1 Fig — (TIF) [file pone.0135104.s001.tif]

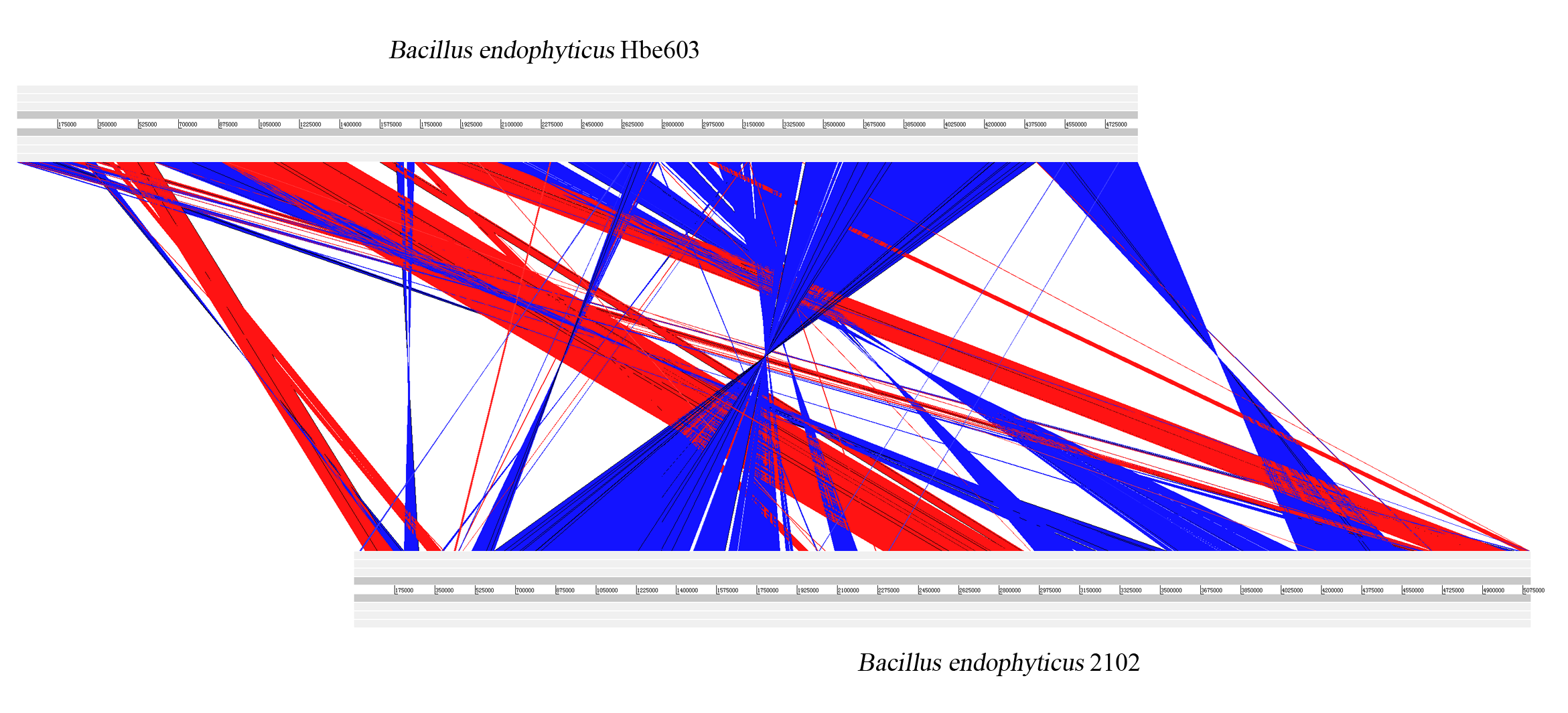

Supplement: S2 Fig — (TIF) [file pone.0135104.s002.tif]
