# Supplementary material for: Genome Sequence of Bacillus endophyticus and Analysis of Its Companion Mechanism in the Ketogulonigenium vulgare-Bacillus Strain Consortium
Source: PLoS One. 2015 Aug 6;10(8):e0135104. doi: 10.1371/journal.pone.0135104 (PMC4527741; doi:10.1371/journal.pone.0135104)
Supplement: S2 Table — (DOC) [file pone.0135104.s004.doc]

**S2 Table. COG category distribution of *B. endophyticus* Hbe603.**

| **COG category** | **Number** | **Content(%)** |
| --- | --- | --- |
| [J] Translation, ribosomal structure and biogenesis | 169 | 4.13 |
| [K] Transcription | 386 | 9.44 |
| [L] Replication, recombination and repair | 130 | 3.18 |
| [D]Cell cycle control, cell division,chromosome partitioning | 31 | 0.76 |
| [V] Defense mechanisms | 63 | 1.54 |
| [T] Signal transduction mechanisms | 174 | 4.25 |
| [M] Cell wall/membrane/envelope biogenesis | 165 | 4.03 |
| [N] Cell motility | 46 | 1.12 |
| [U] Intracellular trafficking, secretion, and vesicular transport | 40 | 0.98 |
| [O] Posttranslational modification, protein turnover, chaperones | 101 | 2.47 |
| [C] Energy production and conversion | 239 | 5.84 |
| [G] Carbohydrate transport and metabolism | 348 | 8.51 |
| [E] Amino acid transport and metabolism | 465 | 11.37 |
| [F] Nucleotide transport and metabolism | 88 | 2.15 |
| [H] Coenzyme transport and metabolism | 164 | 4.04 |
| [I] Lipid transport and metabolism | 155 | 3.79 |
| [P] Inorganic ion transport and metabolism | 272 | 6.65 |
| [Q] Secondary metabolites biosynthesis, transport and catabolism | 125 | 3.06 |
| [B] Chromatin structure and dynamics | 2 | 0.05 |
| [R] General function prediction only | 613 | 14.99 |
| [S] Function unknown | 314 | 7.68 |
