# Supplementary material for: Genome Sequence of Bacillus endophyticus and Analysis of Its Companion Mechanism in the Ketogulonigenium vulgare-Bacillus Strain Consortium
Source: PLoS One. 2015 Aug 6;10(8):e0135104. doi: 10.1371/journal.pone.0135104 (PMC4527741; doi:10.1371/journal.pone.0135104)
Supplement: S3 Table — (DOC) [file pone.0135104.s005.doc]

**S3 Table. Predicted genes related to sporulation in *B. endophyticus* Hbe603.**

| **Stage** | **Gene** | **Annotation** |
| --- | --- | --- |
| Vegetative | *katA* | Vegetative catalase |
|  | *yurY* | Vegetative protein 296 |
| Stage 0 | *kinA* | Sporulation kinase E |
|  | *kinD* | Sporulation kinase D |
|  | *kinE* | Sporulation kinase A |
|  | *spo0A* | Stage 0 sporulation protein A |
|  | *spo0B* | Sporulation initiation phosphotransferase B |
|  | *spo0F* | Sporulation initiation phosphotransferase F |
|  | *spo0H* | Stage 0 sporulation protein A /Sig H |
|  | *spo0J* | Stage 0 sporulation protein J |
|  | *spo0M* | Sporulation-control protein spo0M |
|  | *yaaT* | Stage 0 sporulation protein yaaT |
| Stage II | *spoIIP* | Stage II sporulation protein P |
|  | *spoIIGA* | Sporulation sigma-E factor-processing peptidase |
|  | *spoIIAB* | Anti-sigma F factor |
|  | *spoIIAA* | Anti-sigma F factor antagonist |
|  | *spoIIM* | Stage II sporulation protein M |
|  | *spoIIQ* | Stage II sporulation protein Q |
|  | *spoIID* | Stage II sporulation protein D |
|  | *spoIIR* | Stage II sporulation protein R |
|  | *spoIIE* | Stage II sporulation protein E (Fragment) |
|  | *soj* | Sporulation initiation inhibitor protein soj |
|  | *rsfA* | Prespore-specific transcriptional regulator rsfA |
|  | *ytfJ* | Uncharacterized spore protein ytfJ |
|  | *sigE* | RNA polymerase sporulation mother cell-specific (early) sigma factor SigE |
|  | *yhaL* | Sporulation protein |
| Stage III | *ftsK* | DNA translocase FtsK |
|  | *spoIIIAH* | Stage III sporulation protein AH |
|  | *spoIIIAG* | Stage III sporulation protein AG |
|  | *spoIIIAF* | Stage III sporulation protein AF |
|  | *spoIIIAE* | Stage III sporulation protein AE |
|  | *spoIIIAD* | Stage III sporulation protein AD |
|  | *spoIIIAC* | Stage III sporulation protein AC |
|  | *spoIIIAB* | Stage III sporulation protein AB |
|  | *spoIIIAA* | Stage III sporulation protein AA |
|  | *spoIIID* | Stage III sporulation protein D |
|  | *yunB* | Sporulation protein yunB |
|  | *nucB* | Sporulation-specific extracellular nuclease |
|  | *sigG* | RNA polymerase sporulation forespore-specific (late) sigma factor SigG |
|  | *sigF* | RNA polymerase forespore-specific (early) sigma factor SigF |
| Stage IV | *spoIVA* | Stage IV sporulation protein A |
|  | *spoIVB* | SpoIVB peptidase |
|  | *spoIV* | Putative stage IV sporulation protein |
|  | *safA* | SpoIVD-associated factor A |
|  | *spoIVFB* | Stage IV sporulation protein FB |
|  | *spoIVFA* | Stage IV sporulation protein FA |
|  | *ytrI* | Sporulation membrane protein ytrI |
|  | *sigK* | RNA polymerase sporulation-specific sigma factor (sigma-K) (N-terminal half) |
| Stage V | *spoVR* | Stage V sporulation protein R |
|  | *spoVK* | Stage V sporulation protein K |
|  | *spoVS* | Stage V sporulation protein S |
|  | *spoVFB* | Dipicolinate synthase, B chain |
|  | *spoVFA* | Dipicolinate synthase, A chain |
|  | *spoVAF* | Stage V sporulation protein AF |
|  | *spoVAEA* | Stage V sporulation protein AE |
|  | *spoVAEB* | Stage V sporulation protein AEB |
|  | *spoVAD* | Stage V sporulation protein AD |
|  | *spoVAC* | Stage V sporulation protein AC |
|  | *spoVAB* | Stage V sporulation protein AB |
|  | *spoVAA* | Stage V sporulation protein AA |
|  | *spoVB* | Stage V sporulation protein B |
|  | *spoVT* | Stage V sporulation protein T |
|  | *spoVG* | Putative septation protein spoVG |
| Maturation | *spoVIF* | Sporulation-specific transcription factor spoVIF |
|  | *spoVID* | Stage VI sporulation protein D |
|  | *spmB* | Spore maturation protein B |
|  | *spmA* | Spore maturation protein A |
|  | *cotF* | Spore coat protein F |
|  | *cotA* | Spore coat protein A |
|  | *cotE* | Spore coat protein E |
|  | *cotD* | Spore coat protein D |
|  | *yheD* | Endospore coat-associated protein yheD |
|  | *yheC* | Endospore coat-associated protein yheC |
|  | *yutH* | Endospore coat-associated protein YutH |
|  | *yhcQ* | Spore coat protein F-like protein YhcQ |
|  | *gerQ* | Spore coat protein gerQ |
|  | *sasP-2* | Small, acid-soluble spore protein 2 |
|  | *SASP-C2* | Small, acid-soluble spore protein C2 |
|  | *SASP-C3* | Small, acid-soluble spore protein C3 |
|  | *SASP-C4* | Small, acid-soluble spore protein C4 |
|  | *sspH1* | Small, acid-soluble spore protein H 1 |
|  | *sasP-1* | Small, acid-soluble spore protein 1 |
|  | *tlp* | Small, acid-soluble spore protein tlp |
|  | *sspN* | Small, acid-soluble spore protein N |
|  | *sspI* | Small, acid-soluble spore protein I |
|  | *sspK* | Small, acid-soluble spore protein K |
|  | *yabQ* | Spore protein yabQ |
|  | *yabP* | Spore protein yabP |
|  | *yabG* | Sporulation-specific protease yabG |
| Germination | *gerPE* | Probable spore germination protein gerPE |
|  | *gerPD* | Probable spore germination protein gerPD |
|  | *gerPC* | Probable spore germination protein gerPC |
|  | *gerPB* | Probable spore germination protein gerPB |
|  | *gerPA* | Probable spore germination protein gerPA |
|  | *gerQB* | Spore germination protein gerQB |
|  | *gerQC* | Spore germination protein gerQC |
|  | *gerQA* | Spore germination protein gerQA |
|  | *gerKA* | Spore germination protein KA |
|  | *gerKC* | Spore germination protein KC |
|  | *gerKB* | Spore germination protein KB |
|  | *gerAA* | Spore germination protein A1 |
|  | *gerAB* | Spore germination protein A2 |
|  | *gerAC* | Spore germination protein A3 |
|  | *lipC* | Spore germination lipase lipC |
|  | *gerM* | Spore germination protein gerM |
|  | *gerT* | Spore germination protein gerT |
|  | *gerE* | Spore germination protein gerE |
|  | *gerD* | Spore germination protein gerD |
|  | *yaaH* | Spore germination protein yaaH |
|  | *sleB* | Spore cortex-lytic enzyme |
|  | *csgA* | Sigma-G-dependent sporulation-specific SASP protein |
|  | *cwlC* | Sporulation-specific N-acetylmuramoyl-L-alanine amidase |
|  | *ykvU* | Sporulation protein ykvU |
|  | *ypeB* | Sporulation protein ypeB |
|  | *ydhD* | Putative sporulation-specific glycosylase ydhD |
|  | *paiA* | Protease synthase and sporulation negative regulatory protein PAI 1 |
| Unclassified | *paiB* | Protease synthase and sporulation protein PAI 2 |
|  | *splB* | Spore photoproduct lyase |
|  | *sirA* | Sporulation inhibitor of replication protein |
|  | *sda* | Sporulation inhibitor |
|  | *yjcA* | Sporulation protein yjcA |
|  | *ylbJ* | Sporulation integral membrane protein ylbJ |
|  | *ytrH* | Sporulation membrane protein ytrH |
|  | *cse60* | Sporulation protein cse60 |
|  | *whiA* | Putative sporulation transcription regulator WhiA |
|  | *ydcC* | Sporulation protein ydcC |
